# iCAGES: integrated CAncer GEnome Score for comprehensively prioritizing driver genes in personal cancer genomes

**DOI:** 10.1186/s13073-016-0390-0

**Published:** 2016-12-22

**Authors:** Chengliang Dong, Yunfei Guo, Hui Yang, Zeyu He, Xiaoming Liu, Kai Wang

**Affiliations:** 1 Zilkha Neurogenetic Institute, University of Southern California, Los Angeles, CA 90089 USA; 2 Biostatistics Graduate Program, Department of Preventive Medicine, University of Southern California, Los Angeles, CA 90089 USA; 3 Neuroscience Graduate Program, University of Southern California, Los Angeles, CA 90089 USA; 4 Department of Computer Science, New York University, New York, NY 10012 USA; 5 Human Genetics Center, The University of Texas Health Science Center at Houston, Houston, TX 77030 USA; 6 Division of Epidemiology, Human Genetics and Environmental Sciences, The University of Texas Health Science Center at Houston, Houston, TX 77030 USA; 7 Institute for Genomic Medicine, Columbia University, 630 W. 168th St, Room 11-451, New York, NY 10032 USA

**Keywords:** Cancer genomics, Machine learning, Precision medicine, Precision oncology, TCGA

## Abstract

Cancer results from the acquisition of somatic driver mutations. Several computational tools can predict driver genes from population-scale genomic data, but tools for analyzing personal cancer genomes are underdeveloped. Here we developed iCAGES, a novel statistical framework that infers driver variants by integrating contributions from coding, non-coding, and structural variants, identifies driver genes by combining genomic information and prior biological knowledge, then generates prioritized drug treatment. Analysis on The Cancer Genome Atlas (TCGA) data showed that iCAGES predicts whether patients respond to drug treatment (*P* = 0.006 by Fisher’s exact test) and long-term survival (*P* = 0.003 from Cox regression). iCAGES is available at http://icages.wglab.org.

**Electronic supplementary material:**

The online version of this article (doi:10.1186/s13073-016-0390-0) contains supplementary material, which is available to authorized users.

## Background

Cancer carries somatic mutations acquired during the lifetime of an individual [1]. While the majority of these are “passengers”, which are mutated randomly and functionally neutral, a small proportion are “drivers”, which are causally implicated in oncogenesis [2]. When it comes to a patient, the challenge for his/her molecular diagnosis and treatment lies in rapid and accurate identification of these driver mutations from a large amount of background noise from passenger mutations [3, 4], which is important to devise appropriate targeted therapies.

Next-generation sequencing technology has enabled researchers to rapidly identify somatic mutations from a patient by comparing the sequence from his/her tumors with that from blood or other non-cancerous tissues [5]. These mutations have been well-classified, annotated, and visualized by endeavors such as IntOgen-mutations [6]. Accordingly, several other computational tools were developed to help further pinpoint these cancer drivers using readily available personal cancer genomic information or data integrated from these public databases [7–9]. Such tools can be classified into three categories based on the different information they use to identify drivers. The first category uses genomic mutations, the second category uses transcriptomic information, and the third category uses post-transcriptomic information.

The first category of tools, which focuses on genomic mutations, can be further classified into two subcategories: tools for batch analysis and tools for personalized analysis. Some batch analysis tools prioritize genes, such as MutSigCV [10], MuSiC [11], and Youn-Simon [12], while others prioritize different kinds of mutations. For example, computational tools such as CHASM [7], Mutation Assessor [13], and FATHMM (for cancer) [14] prioritize point-coding mutations, whereas FunSeq2 [15] prioritizes non-coding mutations. While these tools paved the way for cancer driver prioritization, there is still significant room for improvement. For example, Gnad et al. [16] found that many current methods or combination of methods for mutation prioritization fail to exceed 81% accuracy in detecting real cancer driver mutations. Mutation prioritization tools for personal cancer genomes, on the other hand, are extremely underdeveloped. Phen-Gen is one of the few tools for prioritizing personal disease driver genes using only mutations identified from next-generation sequencing [17]. Similarly, wANNOVAR/Phenolyzer is a combination of tools that allows identification of disease genes from genotype and phenotype information [18, 19]. However, these tools are general disease gene prioritization tools, rather than focusing on cancer, and may not work well for cancer somatic mutations due to the use of an allele frequency model from germline mutations. A more detailed comparison of the many tools is given in Table 1.

**Table 1:** Functionality comparison between iCAGES and other cancer driver gene detection tools. "V" represents "available"

Category	Sub category	Sub Subcategory	Output	Tool	VCF input format	Single patient analysis	Structural variation analysis	Web server	Personalized drug	Graphical result	Prior knowledge integration	Non-coding Variant
Genomic variant analysis tools	Batch analysis tools	Driver point mutation prioritization	Protein-coding drivers	CHASM		**V**		**V**				
				Mutation Assessor		**V**		**V**				
				FATHMM		**V**		**V**				
				SIFT		**V**		**V**				
				PolyPhen-2		**V**		**V**				
				GERP++		**V**						**V**
				VEST	**V**	**V**		**V**				
			Non-coding drivers	FunSeq2		**V**		**V**				**V**
		Driver gene prioritization		MuSiC			**V**					**V**
				MutSigCV			**V**	**V**				**V**
				Youn-Simon				**V**				**V**
	Personal analysis tools	Driver gene prioritization		**iCAGES**	**V**	**V**	**V**	**V**	**V**	**V**	**V**	**V**
				Phen-Gen	**V**	**V**		**V**			**V**	
				OncoIMPACT		**V**	**V**					
Transcriptomic expression analysis tools				PARADIGM-SHIFT			**V**					
				DawnRank		**V**						
				CONEXIC			**V**					
				TieDIE								
				DriverNet			**V**					
				Memo			**V**				**V**	
				Dendrix			**V**					
Phosphorylation analysis tools				ActiveDriver								**V**

Besides computational tools that use genomic mutations as input, other tools use transcriptomic or post-transcriptomic information as input. Some tools, such as PARADIGM-SHIFT [20], DawnRank [21], OncoIMPACT[22], and ActiveDriver [23], provide personal cancer driver gene prediction. However, they require gene expression, phosphorylation, or copy number variation data from patients, all of which are not often feasible to obtain due to cost and other practical issues. Moreover, they require complicated data preprocessing and data transformation, which represent challenges for average biologists and clinicians (Table 1).

Thus, there is a strong need for a robust and user-friendly tool to systematically predict personal cancer drivers, which motivated us to develop iCAGES. For an individual patient with cancer, iCAGES takes his/her somatic mutation profile as input and rapidly prioritizes cancer driver mutations, genes, and targeted drugs. iCAGES consists of three consecutive layers. The first layer prioritizes personalized cancer driver mutations, including coding mutations, non-coding mutations, and structural variations. The second layer links these mutation features to genes using a statistical model with prior biological knowledge on cancer driver genes for specific subtypes of cancer. The third layer better serves clinicians and researchers interested in personalized cancer therapy, generating a prioritized list of drugs targeting the repertoire of these potential driver genes. iCAGES can help increase the accuracy of cancer driver detection and prioritization, bridge the gap between personal cancer genomic data and prior cancer research knowledge, and facilitate cancer diagnosis and personalized therapy.

## Methods

### Training data composition

Two types of training datasets were collected for training radial support vector machine (SVM) variants and iCAGES gene scores, respectively. For training the radial SVM, we retrieved a benchmarking dataset from the Martelotto et al. [24] benchmarking study. We annotated all mutations using ANNOVAR [25] with 11 predictors of interest, including SIFT [26], PolyPhen-2 [27], LRT [28], MutationTaster [29], Mutation Assessor [13], FATHMM [30], GERP++ [31], PhyloP [32], CADD [33], VEST [34], and SiPhy [35]. We used training data from 805 functionally validated cancer-related missense mutations as true positive (TP) observations and 134 functionally neutral missense mutations as true negative (TN) observations. For training the logistic regression (LR) for calculating the iCAGES gene score, we collected 6971 mutated genes from 963 breast cancers from the The Cancer Genome Atlas (TCGA) data as a training dataset [36]. A total of 819 genes, which are either significantly mutated genes (SMGs) in the TCGA Pan-Cancer cohort [37] or genes in the Cancer Gene Census [38], were defined as TP observations. The remaining 6152 genes were defined as TN observations. To avoid potential false negative observations in our TN training set, we required that all TN observations not be present in the KEGG Cancer Pathway [39], or be defined as Oncogenes by the UniProt database [40], or be defined by the Cancer Suppressor Genes in the TSGene database (https://bioinfo.uth.edu//TSGene1.0/index.html) [41], or have a maximum radial SVM score greater than 0.93. Next, for each observation, we harvested their four feature scores as follows. For the radial SVM score, we trained the radial SVM model using 939 point coding mutations and calculated the predicted radial SVM score for each point coding mutation in each of the 6971 mutated genes in the training dataset for the iCAGES gene scores (default model). Additionally, to accommodate users who are interested in differences in driver gene prioritization in different cancer subtypes, we also trained a complementary model for all cancer subtypes (Additional file 1: Table S1). We downloaded data for all 14,169 cancer patients from TCGA data portal (August 2016), used Cancer Gene Census genes as TP observations for each cancer subtype and all other mutated genes as TN observations, and trained cancer subtype-specific models for each cancer subtype.

### Testing data composition

Four types of testing datasets were collected for testing the performance of iCAGES. For testing the radial SVM, we curated two testing datasets. Testing dataset I contains 14,984 non-redundant missense mutations curated from the COSMIC database version 68 (as TP observations) and UniProt database (as TN observations) [42, 43]. The rationale for using the COSMIC database to construct our TP dataset was similar to what was used in the FATHMM study [14, 30]: frequencies of these mutations in the database are likely to reflect the importance of these mutations in cancer. We used 14,984 point coding mutations from the COSMIC version 68 database and UniProt database as a benchmarking dataset [42, 43]. A total of 9574 non-redundant somatic point coding mutations obtained from the COSMIC database were used as TP observations, with the following filtering criteria: first, all mutations should be somatic cancer mutations and identified through whole genome-wide sequencing studies; second, all mutations should be recurrent mutations (occurrence greater than or equal to 4); third, all mutations should have a maximum minor allele frequency (MMAF) among population of 0.01 or less (the MMAF is from various populations obtained from three major sources, NHLBI Go ESP for all subjects, African Americans, and European Americans [44], 46 unrelated subjects sequenced by the Complete Genomics [45], and the 1000 Genomes Project for all subjects, admixed Americans, Europeans, Asians, and African populations [46]); fourth, all mutations should have no missing values for all predictors of interest, including SIFT [26], PolyPhen-2 [27], LRT [28], MutationTaster [29], Mutation Assessor [13], FATHMM [30], GERP++ [31], PhyloP [32], CADD [33], VEST [34], and SiPhy [35]. A total of 5410 neutral point coding mutations obtained from the UniProt database were used as TN observations, with the following filtering criteria: first, all mutations were annotated as not known to be associated with any phenotypes, based on the UniProt annotation; second, all mutations have a population MMAF greater than or equal to 0.20; third, all mutations in the TN dataset should not overlap mutations in the TP dataset—if they did, they were removed from the TP dataset. To evaluate the performance of iCAGES for identifying cancer driver mutations from passengers in cancer driver genes, we curated testing dataset II, which contains a subset of all variants in cancer driver genes in testing dataset I.

For batch analysis of the iCAGES gene score, we curated five testing datasets. Testing dataset I contains data for all 14,169 patients from 35 subtypes, all downloaded from the most current version of TCGA data portal (https://gdac.broadinstitute.org; downloaded on August, 2016). We define TP observations as genes in the Cancer Gene Census database and TN observations as mutated genes that are not included in it. Testing dataset II contains molecular profiles of 6748 cancer samples used in Rubio-Perez et al. [47], with which the driver database IntOgen was generated. Testing dataset III contains molecular profiles of 3178 TCGA cancer patients used in the Kandoth et al. study [37]. Since testing dataset III contains patients whose data was also used in testing dataset II, which may inflate the performance evaluation of IntOgen, we generated testing dataset IV, which contains 71 unique cancer patients from testing dataset III but not from testing dataset II. Testing dataset V contains a TCGA mutation dataset used by Kandoth et al. [37] that is independent of TCGA mutation dataset used for training iCAGES gene score. The data for testing dataset V were downloaded from the original publication and analyzed through the iCAGES pipeline for annotation and prediction. Note that to maintain consistency, the same process for filtering and defining TP and TN observations was used as when creating the training dataset. For personalized analysis, mutation data for two patients were downloaded from the original publications from Imielinski et al. [48] and Wagle et al. [49], parsed and analyzed through the iCAGES pipeline.

The last type of testing dataset is the TCGA targeted therapy patient cohort to validate the performance of the iCAGES drug searching module (Additional files 2, 3, and 4). We curated three testing datasets for this purpose. Testing datasets I and II contain two major components, genomic variants and clinical information. For genomic variants, we used TCGA Assembler and downloaded all copy number variation (CNV) data for these patients; we also wrote our own TCGA data portal web crawler and downloaded all remaining single-nucleotide variants and indels for them [50]. For clinical information, we also used TCGA Assembler and downloaded all patients’ clinical and demographic information; we used customized scripts to parse and process these data to link each patient’s clinical data with his/her own genomic variant information. The only difference between testing datasets I and II is that the former contains only individuals that also appeared in Rubio-Perez et al. Testing dataset III is the same as in Rubio Perez et al. to benchmark the performance difference from iCAGES.

To test the performance of the drug nominating layer of iCAGES, we compiled a list of drugs that comply with FDA targeted drug prescription guidelines. We carefully read the FDA label for each of these drugs and made sure that each is currently approved by the FDA and can be used for treating certain cancer subtypes. Moreover, as iCAGES only handles somatic mutation profiles, we required these FDA targeted drugs to specify at least one type of genetic variant testing, which should not be chromosome rearrangement (since this is challenging to determine given the patients’ exonic somatic mutation profiles provided by TCGA). We require that each patient has the same cancer subtype as the FDA drug is approved for and has the particular mutation that the drug requires testing for to include this FDA drug in a patient’s recommended FDA targeted therapy. We found six patients that are eligible for such an FDA drug, two of whom suffered from progressive disease and the remaining four have unknown response. We also compiled a list of drugs for each patient using DGIdb to compare its performance with that of iCAGES. To compile these drugs, we require that they directly target mutations harbored in the patient and we use the whole report without any filtering criteria from the DGIdb API.

### Structural variants, small insertion/deletions, and non-coding variants

For structural variants, we developed a normalized signal score for assessing their cancer driving potential. For potential loss-of-function mutations, the proxy scores, namely CNV normalized signal scores, are the normalized recurrent focal CNV deletion density signal scores retrieved from Kim et al. [51] among cancer suppressor genes classified by Zhao et al. [41]. In the original publication of Kim et al., every CNV region was assigned a focal recurrent amplification/deletion signal, which was calculated based on extensive computational analysis of 8227 copy number profiles gathered from 107 studies on human cancer genomes. Given the somatic mutational profile of a cancer patient, iCAGES extracts structural variants from the mutational profile and searches in the database to see whether or not these structural variants occur in the recurrent focal amplification/deletion regions. If variants do occur within these regions, iCAGES then uses a regional annotation module to assign the corresponding recurrent amplification or deletion signals to each one of the variants as their CNV density; for variants that do not occur in these regions, we assign 0 as the CNV density score. With the same rationale, for gain-of-function mutations we used normalized recurrent focal CNV duplication density signal scores retrieved from Kim et al. [51] among oncogenes in the UniProt database [40]. To annotate a given structural variant, therefore, we first filtered all potential loss-of-function/gain-of-function mutations harbored in these putative cancer suppressor genes/oncogenes and then annotated them with the corresponding normalized CNV signal scores to estimate their cancer driving potential (Additional file 1: Figure S1). To annotate small insertions and deletions given limited knowledge of their mode of action, we assigned each variant to be both a loss-of-function and gain-of-function mutation for downstream modeling and used the more deleterious annotation score as its final variant score.

For the point non-coding mutation score, we retrieved whole-genome FunSeq2 scores (http://FunSeq2.gersteinlab.org/downloads) and used them to annotate each point non-coding mutation for all genes in the iCAGES gene score training dataset [15]. Note that for a gene harboring more than one mutation of the same category, we used the maximum score of each category as the final feature score for each gene.

### SVM modeling and feature selection for the iCAGES variant score

**Fig. 1:**
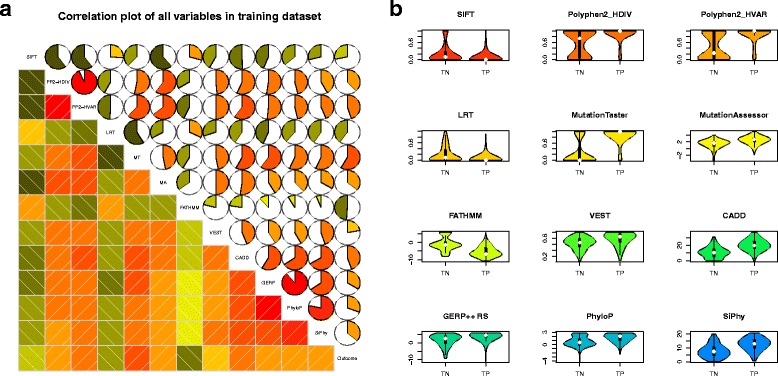
Analysis of each predictor selected for the radial SVM modeling for iCAGES variant score. **a** Correlation diagrams illustrating the pairwise Pearson correlation between all predictors and outcome variable in the training dataset. The *color* and *size* of the shaded region in the pie charts at the *upper right* indicate the level of correlation, with *red* and larger proportions of the shaded region indicating higher positive correlation. **b** Violin plots of scores from different predictors (different colors) in the training dataset in the TP (deleterious) and TN (neutral) groups. Each plot shows the median (indicated by the *small white dot*), the first through the third interquartile range (the *thick*, *solid vertical band*), and the density (different colors) of the predictor scores in each group

Eleven predictors were used for SVM modeling, including SIFT, PolyPhen-2, LRT, MutationTaster, Mutation Assessor, FATHMM, GERP++, PhyloP, VEST, CADD, and SiPhy. The rationale for using these 11 different predictors is described as follows. First, each contributes a different facet of information for a coding variant (Additional file 5). Second, the pairwise Pearson correlation coefficient of these scores also shows that each of these scores provides additional information for describing a coding variant (Fig. 1). Third, our previous study demonstrated the benefit of integrating nine of these predictors into a single model to boost the prediction power in Mendelian disease studies [52]. Here, we used a similar approach for cancer studies, hoping to boost the predictive power of cancer driver variants. Of these, SIFT, PolyPhen-2, LRT, MutationTaster, Mutation Assessor, and FATHMM were transformed to a 0–1 scale using the same methods described in dbNSFP [53, 54], with 1 indicating the highest potential of deleteriousness. Violin plots of all predictors were analyzed to determine whether there were any outliers that were biologically infeasible and to roughly investigate their distributions in the TP and TN observations. Pairwise Pearson correlation coefficients of all continuous and binary variables were calculated to examine potential collinearity between predictor variables and to roughly assess the predictive power of each predictor. Since we observed strong collinearity between HumDiv- and HumVar-trained PolyPhen-2 predictors, we chose HumDiv data as recommended by the developers.

For TP observations, we required that their MMAF be less or equal to 0.01 to filter for somatic mutations that were actually germline mutations. For TN observations in our data, we required all mutations to have a MMAF greater than or equal to 0.20 to filter for mutations that frequently occurred in the population and were therefore likely to be neutral polymorphisms. Such filtering was realized using ANNOVAR with the proper PopFreqMax parameter settings [25].

To test the hypothesis that a non-linear combination of predictors can better model the patterns of cancer driver mutations, two linear machine-learning algorithms, including LR and linear SVM, and a non-linear algorithm, radial SVM, were evaluated using the R package “e1071” [55]. After the radial SVM was selected to model the patterns of cancer driver mutations, its parameters were further tuned to enhance its performance. In the radial SVM, γ measures the size of the radial kernel $\left({\mathrm{e}}^{{}^{\hbox{-}}\gamma}\left|{}^{\mathrm{u}\hbox{-} \mathrm{v}}\right|{}^2\right)$: the larger the γ, the smaller the size of the kernel. And the parameter c is a constant of the regularization term in the Lagrange Formulation that measures the cost of constraint violation: the larger the c, the more cost for constraint violation. These two parameters were tuned and the best set of parameters with the least potential of overfitting was used in the final model. Backward feature selection was also applied in the model to select a parsimonious set of predictors. Diagnostic plots for all models were examined and all model assumptions were evaluated in the LR model. The predictive performance of all models was evaluated using the receiver operating characteristic (ROC) curve with a five-fold cross-validation. For each ROC curve, a 95% confidence interval (CI) was calculated with 2000 bootstrap replicates implemented using the R packages “pROC” [56] and “ROCR” [57], respectively.

To select the proper predictors for establishing the radial SVM, we investigated the correlation between every single predictor with cancer pathogenicity using data in our training set. From the result, individual predictors, including SIFT, PolyPhen-2 (trained on HumDiv), LRT, MutationTaster, Mutation Assessor, FATHMM, GERP++, SiPhy, VEST, CADD, and PhyloP scores all demonstrated high linear correlation with point coding mutations being cancer pathogenic; therefore, they were selected for radial SVM modeling (*P* < 0.0001 with Bonferroni correction). Moreover, no pair of predictors had collinearity issues, so all predictors can be used in one model without encountering potential numerical problems. In addition, the distribution of each individual predictor among the TP and TN observations demonstrated distinctive differences, especially for PolyPhen-2, MutationTaster, and SiPhy, further justifying the use of these predictors for modeling (Fig. 1; Additional file 6).

To test whether we can further enhance the performance of the radial SVM in distinguishing TP from TN observations, we also performed backwards model selection for selecting the best cocktail of predictors, using the area under the curve (AUC) value from the ROC curve as criterion. Results showed that the full model containing all 11 predictors performs the best with the highest AUC value and was thus chosen to be the preliminary model (AUC = 0.89, 95% CI 0.85–0.93; Additional file 1: Figure S2; Additional file 7 and Additional file 8). We also tuned our parameters in the preliminary model to further improve its performance. The combination of c = 10 and γ = 0.001 achieved good performance with intermediate cost and γ and was chosen to be the set of parameters for the final model. Therefore, our final radial SVM model measuring the cancer pathogenicity of every single point coding mutation in a personal genome included all 11 predictors with parameter cost = 10 and γ = 0.001.

### LR modeling for the iCAGES gene score

We constructed a LR model for each gene as follows. First, we examined summary statistics for all four feature variables, including three output scores from the first layer and Phenolyzer score retrieved from the Phenolyzer web application. This step is to ensure that the use of all four feature variables is valid. Moreover, the distributions of all of these variables were analyzed to determine whether there are any outliers that were biologically infeasible. Pairwise Pearson correlation coefficients of all continuous and binary outcome variables were calculated to check potential collinearity between the predictor variables and to investigate the unadjusted relationship between the outcome and each predictor. No collinearity was observed between predictors using R-square (square of Pearson correlation coefficient) of 0.90 as the criterion; therefore, all predictors were used for modeling potential cancer-driving genes, using multiple LR (Additional file 7). Second, after justifying the validity of all four variables, we used them and fitted a non-regularized multiple LR model in R with the following optimization function.

$$ \underset{w,b}{ \min \frac{1}{m}}{\displaystyle \sum_n}\left(-{y}_n \log \left(h\left({x}_n\right)\right)-\left(1-{y}_n\right) \log \left(1-h\Big({x}_n\right)\right) $$

$$ h\left({x}_n\right)=\frac{1}{1+{e}^{-\left[{w}^T{x}_n+b\right]}} $$

where y_n_ denotes the binary outcome measuring the pathogenicity of the *n*th gene, x_n_ is a column vector containing all four predictor scores for the *n*th gene, w is a column vector of weights assigned to every predictor score, b is the bias term (or constant) in the linear combination of predictors, and h(x_n_) is the sigmoid function of x_n_.

The reason for using a non-regularized model is because the number of variables is very small and therefore over-fitting is unlikely to be an issue. We outputted weights of all four predictors generated in the LR model in Additional file 9 and used them as a template to calculate LR predicted cancer driving probabilities, namely h(x_n_), for any given mutated gene in a cancer patient.

### Feature construction for the iCAGES drug scores

One criterion used to rank drugs is to examine the activities on their targets. Recent large-scale drug screening programs, such as the NIH Molecular Library Program, screen for drugs that modulate the activity of gene products and provide their bioactivity score by measuring the activity of each drug in bioassays. Available in the PubChem database, such bioactivity scores are collected and averaged over each drug to estimate average marginal activities [58]. To measure drug activity, we retrieved PubChem PCAssay Data from the PubChem (ftp://ftp.ncbi.nlm.nih.gov/pubchem/Bioassay/CSV/Data/). PubChem activity scores were averaged over each drug and their distributions examined and potential outliers were removed. Next these scores were normalized to a 0–1 scale and used as PubChem active probability for each drug. On the other hand, it is known that drugs can also indirectly interact with a gene. We reason that, by including drugs that target neighbors of the mutated genes in the patient, we can broaden the scope of personalized cancer drug discovery and increase the patient’s chance of getting proper treatment. To measure relatedness of each mutated gene with its neighbor, we retrieved raw BioSystems relatedness scores from the BioSystems database (ftp://ftp.ncbi.nih.gov/pub/biosystems/CURRENT), normalized them to a 0–1 scale, and used them as BioSystems relatedness probabilities for each pair of genes. BioSystems is curated by NIH/NCBI and is perhaps the most comprehensive knowledgebase for biological pathways. It is an aggregated database of several source databases: KEGG, BioCyc (including its tier 1 EcoCyc and MetaCyc databases and its tier 2 databases), Reactome, the National Cancer Institute’s Pathway Interaction Database, WikiPathways, Genome Oncology, and others.

Mining for targeted therapies can be improved if the functions of their targets are known. We incorporated prior knowledge and functionally annotated each gene to be “cancer suppressors”, “oncogenes”, or “other genes” [41, 59]. It is known that cancer is a Darwinian process played out in somatic tissues, so to search for effective drugs for patients, we focused on drugs that can potentially disrupt the evolutionary advantage caused by mutated genes in cancer tissue. For example, if a cancer patient harbors a mutated MTOR, which is an oncogene, then we should search for drugs that negatively influence its function. Likewise, for mutated cancer suppressor genes, we should search for drugs that positively influence the function of this gene. Therefore, for each candidate cancer driver gene predicted in the second layer, iCAGES queries the drug–gene interaction database DGIdb for expert-curated drugs which “activate” tumor suppressors, “inhibit” oncogenes, and interact with other genes.

Given a list of potential cancer driver genes, each with an iCAGES score, we search for targeted drugs as follows. First, to search for their neighboring genes, we query the BioSystems database for each gene and its top four most-related neighbors according to their normalized BioSystems relatedness probability. The rationale is that we want to find drugs that target not only the actual mutated gene but also its close “neighbors” in biological pathways and weight them differently depending on the “distance” between the “neighbors” and the actual mutated gene. This is because, based on our experience, ~50% of the time there is no drug directly targeting the repertoire of mutated genes in a patient. In this case, interrupting the pathway of these genes can be an alternative strategy to find targeted drugs for this patient [49, 60, 61]. Second, we classify each gene to be a cancer suppressor, oncogene, or other type of gene by querying the TSGene database and UniProt oncogenes. Third, we use the DGIdb database to query targeted drugs activating cancer suppressors, inhibiting oncogenes, and interacting with other genes, respectively, with different parameter settings. More specifically, for cancer suppressor genes, we query for expert-curated drugs with positive influence on expression of these genes through the following terms of DGIdb: activator, inducer, positive allosteric modulator, potentiator, and stimulator. For oncogenes, we query for expert-curated drugs with negative influence on expression of these genes through the following terms of DGIdb: agonist, antisense, competitive, immunotherapy, inhibitory allosteric modulator, inverse agonist, negative modulator, partial agonist, partial antagonist, vaccine, inhibitor, suppressor, antibody, antagonist, and blocker. For other genes, we query for expert-curated drugs with any kind of interaction with the target. We also curated a list of FDA-approved cancer therapies to complement DGIdb’s drug collection and searched for drugs in this list that directly or indirectly interact with the set of mutated genes and their neighbors. Finally, for each drug, given the BioSystems relatedness probability of its direct target with the original mutated gene, PubChem active probability for the drug, and iCAGES gene score for the original mutated gene, we can calculate the joint probability of a drug being a therapeutic candidate for the patient by multiplying these three probabilities together, generating an iCAGES drug score.

We also classify each drug to be a first tier drug if they are FDA approved, second tier drug if they are undergoing clinical trials based on active records of clinical trials retrieved from https://clinicaltrials.gov, and third tier drugs if otherwise. We currently consider these following two types of repurposing. First, tumor type repurposing (tumor type different than clinical guidelines). Second, alteration repurposing (drugs targeting a mutation in a driver gene being employed for another mutation in the same gene). For drugs (FDA approved or clinical trial drugs) that fit either one of the repurposing criteria, we include them as tier 1 (for FDA approved drugs) and tier 2 (for clinical trial drugs). As DGIdb drugs are computationally predicted drugs for general gene–drug interaction research and hence lack information on their effective mutations and cancer types, we currently have not designed a repurposing strategy for them.

### Indexing and speed optimization

To speed up large database queries, we created a binary linear indexing and searching algorithm termed TDS (Two-step dictionary searching). Each database is split into many bins of X lines (by default X = 10) for each chromosome/contig. TDS creates a binary index file recording positions of these bins in the database. Meanwhile, TDS maintains a static array of chromosome/contig names and positions of first bins in the index file. During a search, TDS first locates the bin position in the index, then performs a sequential search from the particular bin in the database. Time costs are bounded by two random accesses plus X or fewer sequential accesses. Chromosome/contig name can be generalized to any key name in a key-coordinate structured database; therefore, TDS is not limited to applications in genome studies. Tests on NA12878 whole genome SNP/indel VCF data (more than four million records) from the Genome In a Bottle consortium [62] and FunSeq2 annotation (~200 GB) show that TDS outperforms Tabix [63] by ~30% (Additional file 1: Figure S3).

### Statistical analysis

Statistical analysis and LR modeling was conducted using R (version 3.0.1). The ROC curve was drawn using the R package “ROCR”. Using “pROC” a 95% CI was calculated. SVM modeling was conducted using “e1071”. The iCAGES package was written in Perl and the user interface was written in Ruby on Rails, JavaScript, and HTML5. For survival analysis, we used Stata 14.1 to perform analysis and generate all results and figures.

### Input and output of iCAGES

iCAGES takes somatic mutations from a patient as input. This input file can be in either ANNOVAR [25] input format, VCF format, or BED format. For VCF format, it considers three types of input files. The first type contains only somatic mutations detected in one single patient. The second type contains both tumor and germline mutations in a single patient. This is the raw output file format from several somatic mutation detection tools. The third kind contains both tumor and germline mutations in multiple patients. In the last case, the user needs to specify which sample he/she needs to analyze so that iCAGES can perform a single patient analysis based on the particular patient of interest. For the BED format, it can be used to specify locations where structural variants occur or locations of genes that are over- or under-expressed for advanced users. Another optional input is the subtype of cancer, as specified by the user, which activates iCAGES to apply the models trained for this particular subtype to further enhance its predictive performance. Major output files from iCAGES contain three CSV (comma-separated value) files, each corresponding to results from a layer of iCAGES. The first CSV file contains cancer driver mutation prioritization and includes information such as mutation context, mutation category (in the current study, we classify mutations into three categories, point coding mutations, point non-coding mutations, and structural variations) and driver mutation score. Note that each driver mutation score corresponds to a mutation category. The second CSV file contains cancer driver gene prioritization results and includes information such as gene category (in the current study, we classify genes into three categories, genes in the Cancer Gene Census [64, 65], genes in the KEGG cancer pathway [66], and genes in other categories), maximum radial SVM score, and iCAGES gene score. The third CSV file contains personalized drug prioritization results and includes information such as predicted drugs, their final target, and their iCAGES drug scores (Table 2). To facilitate interactive graphics rendering for iCAGES web server, a JSON file was also generated, which contains the same information as in the three CSV files. Therefore, we have provided information showing the extensive utility of iCAGES for average biologists, clinicians, and patients.

**Table 2:** Major output files of iCAGES

Category	Column name	Description	Example
Mutation prioritization output	Gene name	HUGO name of the gene	ARAF
	Chromosome number	Chromosome number of this mutation	1
	Coordinate	Genomic coordinate of the mutation	1234560
	Reference allele	Reference allele of this mutation	C
	Alternative allele	Alternative allele of this mutation	G
	Mutation category	Whether this mutation is a point coding mutation, point non-coding mutation, or structural variation	Point coding mutation
	Mutation context	Genomic context of the mutation	c.641C > G
	Protein context	Protein context of the mutation	p.S214C
	Score category	If the mutation is a point coding mutation, then the category is the radial SVM score; if a point non-coding mutation, then Funseq2 score; if structural variation, then normalized CNV signal score	Radial SVM
	Driver mutation score	Value of the corresponding score	0.932
Gene prioritization output	Gene name	HUGO name of the gene	ARAF
	Gene category	Whether this gene belongs to the Cancer Gene Census, KEGG Cancer Pathway, or other categories	KEGG cancer pathway
	Maximum radial SVM score	Maximum radial SVM score of all point coding mutations in the gene	0.932
	Maximum FunSeq2 Score	Maximum FunSeq2 score of all point non-coding mutations in the gene	0.000
	Maximum normalized CNV Signal score	Maximum normalized CNV signal score of all structural variations in the gene	0.000
	Phenolyzer score	Phenolyzer score of the gene	0.306
	iCAGES gene score	iCAGES gene score of the gene	0.484
Drug prioritization output	Drug name	Name of the drug	SORAFENIB
	Final target gene	Mutated gene in the patient finally targeted by the drug	ARAF
	Direct target gene	Mutated gene in the patient directly targeted by the drug	ARAF
	iCAGES gene score	iCAGES gene score of the target gene	0.484
	BioSystems normalized Relatedness probability	BioSystems normalized relatedness probability between the direct target of the drug and the target gene	1.000
	PubChem normalized drug active probability	PubChem normalized drug active probability of this drug	1.000
	iCAGES drug score	iCAGES drug score of the drug	0.484
	Tier	Which tier this drug belongs to, whether it is FDA approved (tier 1), undergoing clinical trials (tier 2) or otherwise (tier 3).	1
	Brand name	Commercial brand name of this drug	NEXAVAR
	FDA approved subtype	What cancer subtypes approved by FDA can this drug be applied to	Hepatocellular carcinoma, renal cancer, thyroid cancer
	Clinical trial name	The name of the active clinical trials on this drug	Sorafenib phase II study for Japanese anaplastic or medullary thyroid carcinoma patients
	Clinical trial organization	The organization for this clinical trial	BAYER
	Clinical trial phase	Phase of this clinical trial	2
	Clinical trial URL	URL of this clinical trial	http://clinicaltrials.gov/show/nct02114658

### Versions of the human reference genome

iCAGES supports three versions of human reference genomes, hg18, hg19 (default), and hg38. When using the iCAGES standalone package or web application, users need to specify the version of the human reference genome. All annotation scores from iCAGES originally used hg19 as the reference genome, and we used liftOver to directly convert radial SVM scores, FunSeq2 scores, and CNV normalized signal scores from hg19 coordinates to hg18 and hg38.

## Results

### Overview

**Fig. 2:**
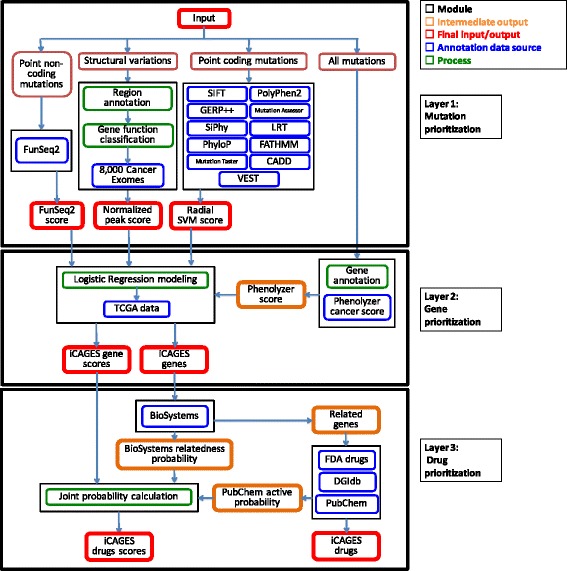
The iCAGES package as three layers. The input file contains all variants identified from the patient; it can be either in ANNOVAR input format or in VCF format. The first layer of iCAGES prioritizes mutations. It computes three different feature scores for annotating the gene, including the radial SVM score for each of its point coding mutations, CNV normalized peak score for each of its structural variations, and FunSeq2 score for each of its point non-coding mutations. The second layer of iCAGES prioritizes cancer driver genes. It takes three feature scores from the first layer, generates the corresponding Phenolyzer score for each mutated gene and computes a LR score for this gene (iCAGES gene score). The final level of iCAGES prioritizes targeted drugs. It first queries the DGIdb and FDA drug database for potential drugs that interact with mutated genes and their neighbors. Next, it calculates the joint probability for each drug being the most effective (iCAGES drug score) from three feature scores, which are iCAGES gene scores for its direct/indirect target, normalized BioSystems probability measuring the maximum relatedness of a drug’s direct target with each mutated gene (final target), and PubChem active probability measuring the bioactivity of the drug. The final output of iCAGES consists of three major elements, a prioritized list of mutations, a prioritized list of genes with their iCAGES gene scores, as well as a prioritized list of targeted drugs with their iCAGES drug scores

A general overview of iCAGES is given in Fig. 2. To prioritize driver mutations, the first layer takes somatic mutations from next-generation sequencing as input and outputs three types of driver potential scores for coding mutations, non-coding mutations, and structural variants, respectively. To prioritize driver genes, the second layer takes two major sources of input. The first source measures the genomic potential of a gene being a personal cancer driver and the second source measures the prior knowledge of a gene being a driver for a specific cancer subtype, based on previous biological knowledge, through Phenolyzer predictions. Given these two sources of input, iCAGES then models the patterns of putative cancer drivers observed in TCGA data with a LR model and outputs a prioritized list of genes ranked by their cancer driving potential, or iCAGES gene scores. To predict personalized treatment, the third layer takes the prioritized list of mutated genes and their iCAGES gene scores, searches for drugs targeting these genes and their neighbors, and prioritizes the drugs according to their pharmacodynamic activities, relatedness of their targets to the mutated genes, and the corresponding iCAGES gene scores. It outputs a prioritized list of drugs ranked by their probabilities of being effective for the particular patient, that is, the iCAGES drug scores. Finally, we implemented iCAGES as a command-line tool and a web server; the latter facilitates users without informatics skills to perform analysis on personal cancer genomes, unlike most other tools (Table 1). In the sections below, we describe the features and performance for each of the three layers in iCAGES and demonstrate the performance of iCAGES using several real-world examples.

### Layer 1: Variant prioritization

#### Description

The first layer is variant prioritization, which tackles three kinds of variants: point coding mutations, point non-coding mutations, and structural variants. For point coding mutations, various tools, such as SIFT [26] and PolyPhen-2 [67], have been developed and widely used; however, they were developed for germline mutations and may not be specifically tuned for cancer somatic mutations. Our previous study has demonstrated the value of integrating multiple scoring methods to enhance the accuracy of classifying Mendelian disease variants [52]. To test whether integrating these methods can also enhance the accuracy of classifying cancer driving variants, we trained a radial SVM model to annotate each point coding mutation based on the scoring patterns of 11 different prioritization tools and evaluated its performance. For point non-coding mutations, we chose FunSeq2 to annotate these mutations because it is currently the only available tool for comprehensively prioritizing cancer drivers for non-coding mutations. Structural variants may cause transcriptional alterations by either loss-of-function or gain-of-function effects and, to our knowledge, there is a paucity of tools for annotating these variants for their cancer driver potentials. Therefore, we developed a method to quantify the cancer driver potential of structural variants based on the property of the structural variants (gain or loss) as well as the cancer relevance of the genes (oncogenes or tumor suppressors) [68]. Since structural variants in cancer tend to be enriched in specific regions in the genome [51], we used a normalized CNV signal score based on CNV duplication and deletion patterns and their presence in oncogenes or tumor suppressors as an estimate of the driver potential of structural variants. Given the limited knowledge on the mode of action of small insertions and deletions, to annotate them we assigned each variant to be both loss-of-function and gain-of-function for downstream modeling and use the more deleterious annotation score for the final variant score.

#### Performance evaluation

**Fig. 3:**
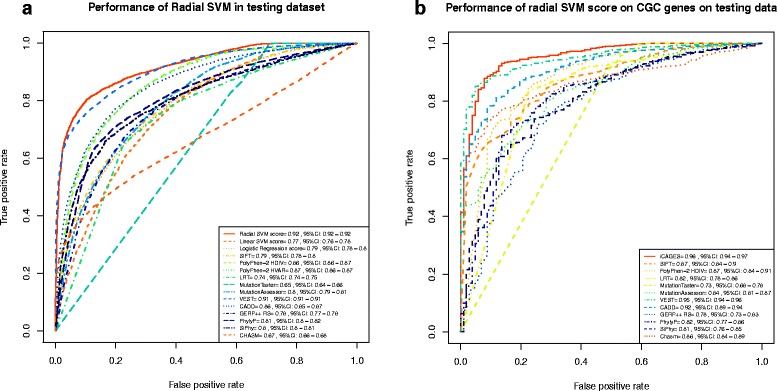
Performance of the first layer of iCAGES. **a** Performance of the radial SVM score evaluated on the COSMIC version 68 testing dataset (testing dataset I). A higher AUC score indicates better performance. The 95% CI was computed with 2000 stratified bootstrap replicates. **b** Performance of the radial SVM score evaluated on Cancer Gene Census genes from COSMIC version 68 testing dataset (testing dataset II)

Among the three mutation feature scores, we highlight the performance of the radial SVM score in the current section. We developed the radial SVM scores using a well curated training data set in the Martelotto et al. [24] benchmarking study and then evaluated its performance on two testing datasets. Testing dataset I contains 14,984 non-redundant missense mutations curated from the COSMIC database version 68 (as TP observations) and UniProt database (as TN observations) [42, 43]. The rationale for using the COSMIC database to construct our TP dataset was similar to that used in the FATHMM study [14, 30]: the frequencies of these mutations in the database are likely to reflect the importance of them in cancer. To evaluate the performance of iCAGES in distinguishing cancer driver mutations from passenger mutations in cancer driver genes, we curated testing dataset II, which contains a subset of all variants in cancer driver genes in testing dataset I. We then measured the performance of different methods using the AUC value of the ROC plot. We found that our radial SVM model performed better than all other variant prioritization tools, including general missense mutation scoring tools and cancer-specific driver mutation detecting tools, in distinguishing drivers in general cases and in cancer driver genes. Among general missense mutation scoring tools, VEST achieved the highest discriminative power (AUC = 0.91, 95% CI 0.91–0.91 for testing dataset I; AUC = 0.95, 95% CI 0.94–0.96 for testing dataset II), which is still slightly lower than our radial SVM model (*P* = 0.01 for testing dataset I, *P* = 0.65 for testing dataset II, one-sided test with 2000 bootstraps) (Fig. 3). Similar results were obtained when comparing our model against the cancer-specific driver mutation detecting tools CHASM [7] and Mutation Assessor [13]. Additionally, the radial SVM score also outperformed other machine learning-based scoring methods, such as linear SVM and LR. For example, even though the linear SVM performed rather impressively (AUC = 0.77, 95% CI 0.76–0.78), it failed to match the radial SVM model (*P* < 1 × 10^−15^ with one-sided test with 2000 bootstraps) (Fig. 3a).

Additionally, we also performed reduced set analysis by training a reduced radial SVM with each feature deleted from the model and compared its performance with the feature that was deleted, further confirming the significant advantage of using radial SVM in predicting drivers (Additional file 1: Figure S4). Therefore, we believe that the radial SVM score proved itself to be a highly effective choice for scoring somatic coding mutations both in general cases and in cancer driver genes in the first layer of iCAGES.

### Layer 2: Gene prioritization

#### Description

The second layer is gene prioritization, which relies on annotation results from the first layer. Harboring variants with different functional effects, the cancer driver potential of each gene can be different depending on the distinct types of mutations, such as coding mutations, non-coding mutations, and structural variants. To model contributions from different types of mutations, we applied a LR model trained on 6971 mutated genes from TCGA data from 963 patients with breast cancer, the most common cancer type in females and one of the most well-studied cancer types [69], using four feature scores described as follows (we wish to stress here that although our default model focused on breast cancer, we do provide models for 35 other subtypes of cancer; Additional file 1: Table S1). To characterize each gene, we annotated each of its mutations with three genomic feature scores: the radial SVM score, CNV normalized signal score, or FunSeq2 score. To link non-coding variants with FunSeq2 score to a gene, we assigned each of such variants to its closest gene in terms of genomic distance. Considering that one gene may harbor more than one mutation of each category, for each gene we took the maximum of each feature score among all mutations and generated its three genomic feature scores. On the other hand, prior knowledge generated from decades of cancer genetic and genomic research can help us improve gene prioritization. Such knowledge is documented in various publically available databases. To quantify such prior knowledge on cancer and its associated genes, we applied a database-mining tool, Phenolyzer, which outputs a ranked list of candidate genes based on their association with specific subtypes of cancer [19]. After integrating these pieces of information, the output of the second layer is the LR-predicted probability for each gene, namely the iCAGES gene score, which measures the cancer-driving potential for this gene. From sensitivity and specificity analysis, we recommend 0.11 as the binary cutoff for the iCAGES gene score (Additional file 1: Figure S5).

#### Performance evaluation

**Fig. 4:**
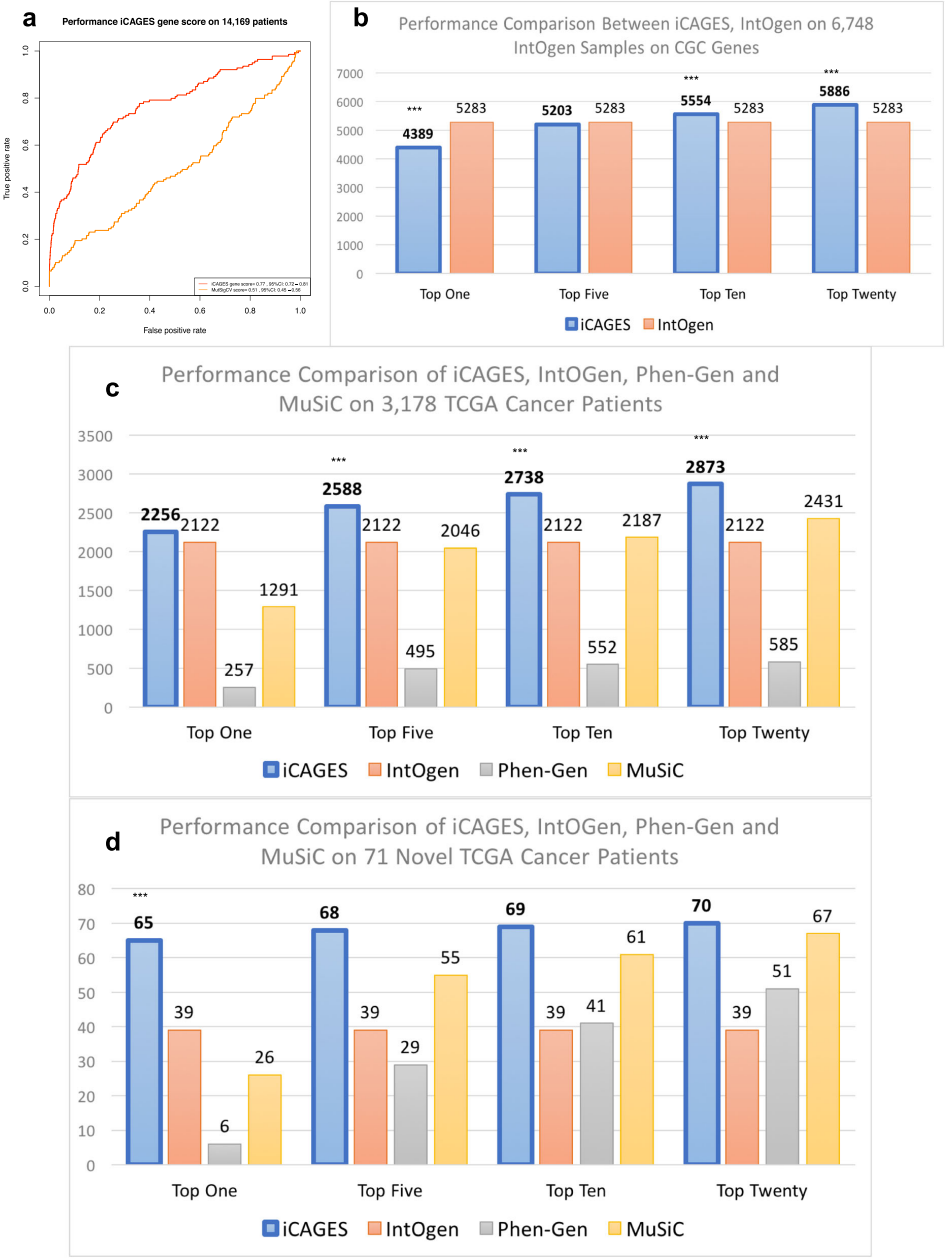
Performance of the second layer of iCAGES. **a** Performance of the iCAGES score compared to MutSigCV, evaluated on 14,169 TCGA patients. A higher AUC score indicates better performance. The 95% CI was computed with 2000 stratified bootstrap replicates (testing dataset I). **b** Performance of iCAGES compared to IntOgen, evaluated on data from 6748 patients used in the Rubio-Perez et al. study. Each bar represents the number of patients whose cancer driver gene can be identified by iCAGES or by IntOgen. *Top One*, *Top Five*, *Top Ten* and *Top Twenty* refer to using the top gene, top five genes, top ten genes, and top 20 genes from prediction, respectively. A significant advantage of iCAGES compared to other tools is indicated with ***P ≤ 0.0001 (Bonferroni correction; testing dataset II). **c** Performance of iCAGES compared to IntOgen, Phen-Gen, and MuSiC evaluated on data from 3178 patients used in the Kandoth et al. study (testing dataset III). **d** Performance of iCAGES compared to IntOgen, Phen-Gen, and MuSiC evaluated on data from 71 patients used in the Kandoth et al. study but not in the Rubio-Perez et al. study (testing dataset IV)

We evaluated the performance of iCAGES on five cohorts of data from cancer patients in large projects or recent publications, as well as on two published cases of targeted therapy-guided exceptional responders, and demonstrated its better performance in all seven cases. Testing dataset I contains molecular profiles from all 14,169 patients from 35 cancer subtypes, all downloaded from the most current version (August 2016) of TCGA data portal. We define TP observations as genes in the Cancer Gene Census database and TN observations as other mutated genes in this cohort. We compared the performance of MutSigCV and iCAGES on this dataset (Fig. 4a) and on each subtype (Additional file 1: Figure S6) and found that iCAGES achieved significantly better performance than MutSigCV in both cases (*P* < 1 × 10^−10^, one-sided test with 2000 bootstraps). Testing dataset II contains 6748 cancer samples used in Rubio-Perez et al. [47], on which driver database IntOgen was generated, to evaluate whether or not iCAGES’ personalized prediction can reach comparable performance with IntOgen’s batch prediction (Fig. 4b). We found that iCAGES did show comparable performance with IntOgen in nominating drivers using the top five genes as candidate drivers and demonstrated significantly better performance using the top 10 or top 20 genes as candidate drivers (*P* < 0.0001, Z-test). Note that IntOgen drivers were generated on this exact dataset using all patients at once while iCAGES only generated personalized output for each cancer patient in the cohort, and yet it achieved comparable or better performance than IntOgen. Testing dataset III contains 3178 TCGA cancer patients used in Kandoth et al.’s study (Fig. 4c). On this dataset we compared the performance of iCAGES against IntoOgen, Phen-Gen, and MuSiC and showed significantly better performance of iCAGES compared to all the other methods, including MuSiC, which was used in Kandoth et al.’s original publication for driver gene analysis (*P* < 0.0001, Z-test). Since testing dataset III contains patients whose data was also used in testing dataset II, which may inflate the performance evaluation of IntOgen, we generated testing dataset IV, which contains 71 unique cancer patients from testing dataset III but not in testing dataset II. In this dataset, we observed a larger proportion of patients with their drivers correctly specified using iCAGES than using IntOgen (1.75-fold change in this testing dataset versus 1.23-fold change in testing dataset III), demonstrating the advantage of iCAGES in driver gene prioritization in new cancer patients. Similar findings were observed in testing dataset V, which used the same source dataset as testing dataset III but differed in the filtering strategy for driver genes (Additional file 1: Figure S7).

Additionally, we compared the performance of the iCAGES gene score with a recently published personal pathogenic gene prioritization tool, Phen-Gen, using data from two recently published cases on personalized cancer therapy. The first case reported the treatment course of a patient with lung adenocarcinoma [48], the most common subtype of non-small cell lung cancer and which remains clinically challenging. Even though recurrent mutations in genes such as *KRAS*, *EGFR*, and *ALK* have been reported, the key oncogenic driver is evasive in most cases [70–73]. Therefore, the study interrogated the cancer genome of this patient, manually examined all mutated genes, and selected one or more potential targets to design personalized therapy accordingly. By investigating the somatic mutations of this patient, Imielinski et al. [48] found that *ARAF* was likely to be one of the cancer driver genes for lung adenocarcinoma in this case and this was responsive to targeted therapy with sorafenib. Using published data of somatic mutations, iCAGES was able to replicate this finding by nominating *ARAF* as the first candidate cancer driver gene out of 129 genes with somatic mutations (Additional file 10). In contrast, Phen-Gen only ranked *ARAF* as the sixth candidate out of 12 genes related to any phenotype accepted by this tool (top 50%) (Additional file 1: Table S12). Similar results were observed in the second case, which was a patient with an advanced solid tumor [49]. While the iCAGES gene score nominated *MTOR* as the third most likely candidate (ranked after *CTNNB1* and *TP53*) out of 649 mutated genes, Phen-Gen did not include *MTOR* as a gene interacting with any phenotype accepted by this tool (Additional file 11, Additional file 1: Table S14). Therefore, we believe that our approach can take into account different effects of different mutations guided by prior knowledge, model the patterns of cancer drivers, and accurately nominate the cancer driver genes.

### Layer 3: Drug prioritization

#### Description

Although the main motivation to develop iCAGES is to identify driver genes from personal cancer genomes, the results may also facilitate the selection of treatment strategies. The third layer of iCAGES aims to link candidate genes to drugs, through a three-step process. The first step queries the BioSystems database for its top neighboring genes in the same biosystem and calculates its normalized relatedness score [74]. The next step classifies each gene into tumor suppressor genes, oncogenes, or other genes and queries the DGIdb database for these different types of genes according to their corresponding cancer evolutionary properties [75]. Additionally, it also queries FDA guidelines and clinical trial databases to include the corresponding targeted drugs, similar to what was used in Rubio-Perez et al. [47]. Moreover, to measure the activity of a drug, we retrieve its marginal activity scores from the PubChem database [76], average them over each drug, and normalize them to 0–1 as the activity score for this drug. The final step calculates the joint probability of a drug being an effective drug for this particular patient by multiplying the iCAGES gene score for its target, its relatedness score, and its marginal activity score, thus generating the iCAGES drug score.

#### Performance evaluation

We tested the performance of this layer on the two aforementioned cases as well as three testing datasets against the Rubio-Perez et al. personalized cancer therapy prescription pipeline [47], DGIdb, and the strict FDA prescription guideline. We demonstrated that the iCAGES drug recommendation pipeline can better leverage predicted driver genes to facilitate the selection of optimal personalized treatment strategies.

For the two case studies, we downloaded the genomic data for these two patients and analyzed them using the iCAGES framework. The first patient, with lung adenocarcinoma, demonstrated an extraordinary response with sorafenib, which was indeed nominated by iCAGES as the top drug candidate out of 122 predicted drugs (Additional file 12). We want to emphasize here that sorafenib is not an FDA-approved drug for treating lung adenocarcinoma; yet iCAGES nominated its off-label use for this patient due to the identification of *ARAF* as a driver gene in layer 2, which was indeed highly effective in this specific patient as reported in the original study. Similarly, for the second patient, with a solid tumor, iCAGES nominated everolimus, which directly targets the *MTOR* gene, as the third best candidate out of 489 predicted drugs (Additional file 13).

**Fig. 5:**
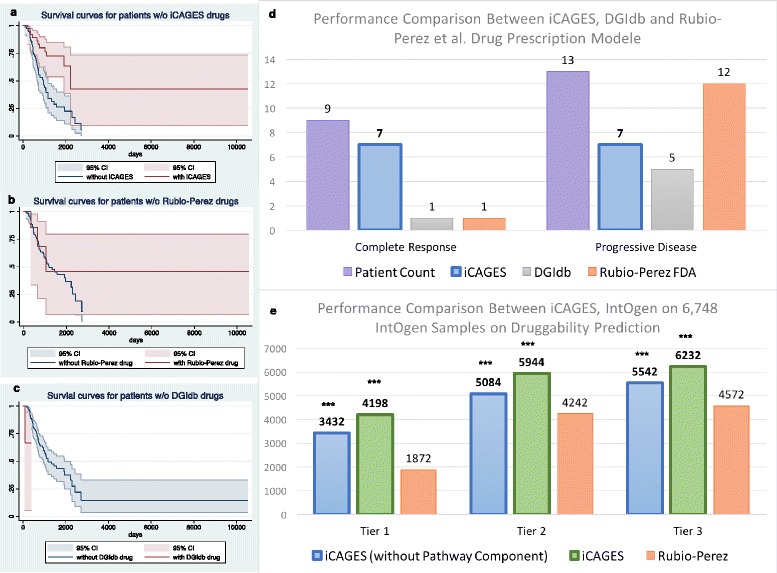
Performance of the third layer of iCAGES. **a**–**c** Kaplan–Meier survival curve for 124 TCGA patients with targeted therapy with unknown response whose data were also used in the Rubio-Perez et al. study (testing dataset I). **a** *Red* and *blue* curves represent patients whose treatments do and do not contain iCAGES-predicted first tier drugs, respectively. *Red* and *blue areas* represent the 95% confidence interval for the survival curve. **b** *Red* and *blue curves* represent patients whose treatments do and do not contain Rubio-Perez et al.-predicted drugs, respectively. **c** *Red* and *blue curves* represent patients whose treatments do and do not contain DGIdb-predicted drugs. **d** Number of TCGA patients with targeted therapy with complete response or progressive disease who received correct iCAGES-predicted drugs (*blue*), DGIdb drugs (*gray*), Rubio-Perez et al. tier one drugs (*orange*). **e** Number of patients used in Rubio-Perez et al. study who can potentially benefit from iCAGES (without pathway component from BioSystem) predicted drugs from three tiers (*blue*), iCAGES-predicted drugs (*green*), Rubio-Perez et al.-predicted drugs (*orange*). Significant advantage of iCAGES compared to other tools is indicated as ***P ≤ 0.0001 and Bonferroni correction (testing dataset III)

For the cohort studies, we complied three testing datasets, on which we evaluated the performance of iCAGES, Rubio-Perez et al.’s strategy, DGIdb, and the strict FDA prescription guidelines. Testing dataset I contains data from 146 patients from TCGA project with annotation on their targeted therapies, which were also used in Rubio-Perez et al.’s study. Among them, 22 have a known response to their therapies. While iCAGES correctly predicted therapies that the complete responders responded to in seven out of nine cases and excluded therapies that incurred progressive disease in seven out of 13 cases, DGIdb correctly predicted six cases in all 22 patients and Rubio-Perez et al. correctly predicted 13 in total (Fig. 5d). For the remaining 124 patients with missing response annotations, we observed 66% higher survival probability for patients whose targeted therapy regime included iCAGES-predicted tier 1 drugs after controlling for age and gender (Cox proportional hazard ratio = 0.34, *P* = 0.002 from Cox regression, 95% CI 0.17–0.66). In comparison, only applying the DGIdb predicted drug (*P* = 0.087 from Cox regression, 95% CI 0.78–46.49) or applying the Rubio-Perez et al. method (*P* = 0.569 from Cox regression, 95% CI 0.22–2.30) did not impact patient survival.

**Fig. 6:**
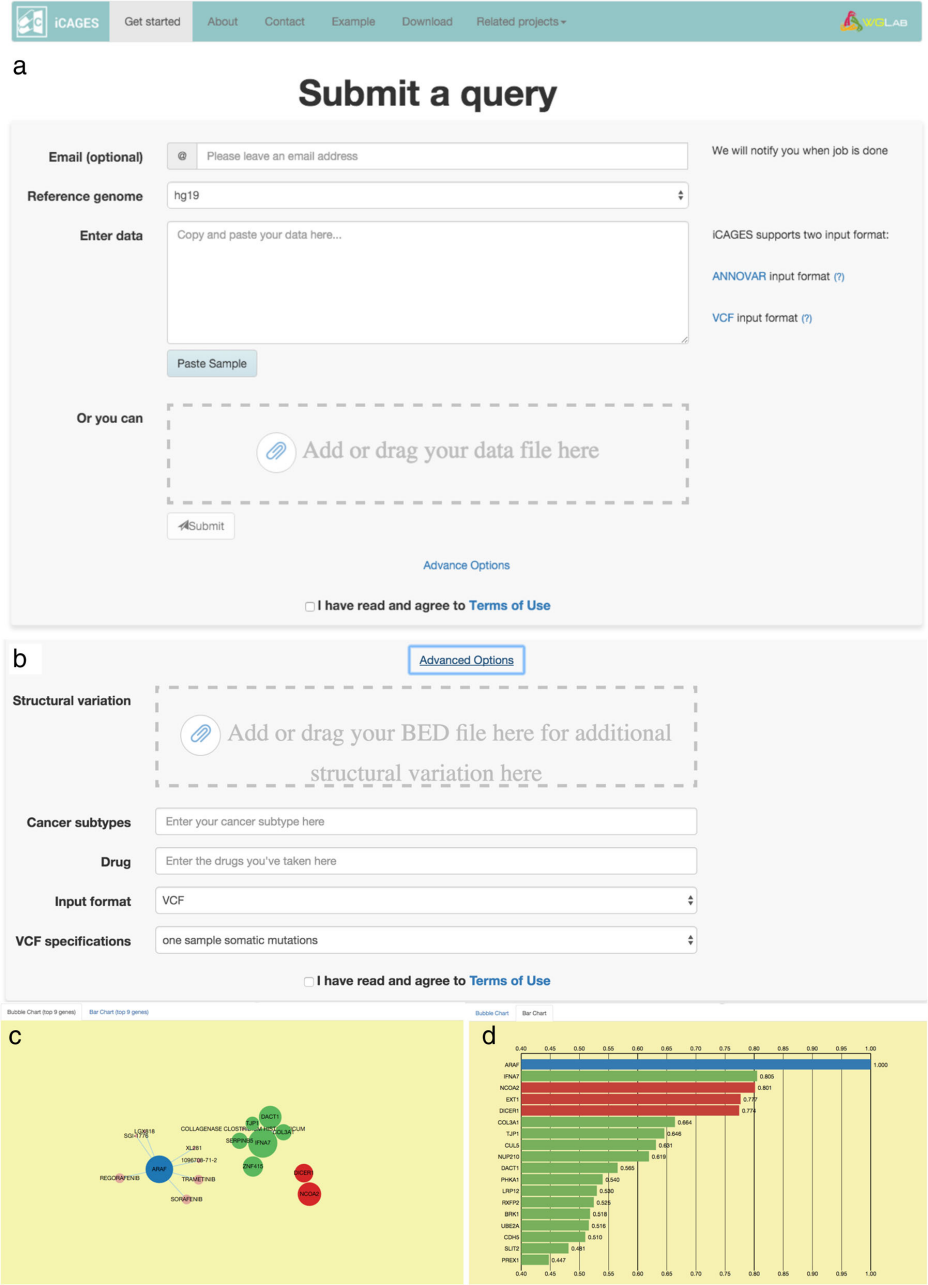
The web interface of iCAGES, as demonstrated using data from Imielinski et al. **a** The submission page for iCAGES. Users can enter data with the VCF format (default) or with ANNOVAR input format used in the ANNOVAR package. **b** Dynamic form for advanced users. Users can click “Advanced Options” and enter additional information, such as structural variations in BED format, cancer subtype, and drugs that this patient has been using. **c** Bubble plot output of the iCAGES package. The size of the bubbles indicates the weight of the iCAGES score and different colors indicate the category of the gene. *Red*, *blue*, and *green* indicate that this gene belongs to the Cancer Gene Census, the KEGG cancer pathway, or neither category, respectively. *Pink* bubbles that are connected to *blue*, *green* or *red* bubbles indicate targeted drugs. **d** The corresponding bar plot of the output. The length of the bar indicates the weight of the iCAGES score and different colors indicate the category of the gene

Similar results were observed on testing dataset II, which contains 335 patients from TCGA project with annotation on targeted therapies. Note that only six patients of this cohort (1.79%) were eligible for using FDA approved drugs according to the FDA prescription guidelines, which was consistent with previous findings. For 68 of them with known responses, iCAGES excluded drugs that incur progressive disease for 52% of the non-responders and predicted drugs with complete response for 15% of the responder group (*P* = 0.006 by two-sided Fisher’s exact test). Moreover, for the remaining 267 patients with unknown response, patients whose targeted therapies include iCAGES-predicted drugs are more likely to survive cancer throughout their lifetime than those whose therapies do not, after controlling for age and gender (Cox proportional hazard ratio = 0.53, *P* = 0.003 by Cox regression, 95% CI 0.36–0.81).

To compare iCAGES’s potential for finding druggable targets, we curated testing dataset III, which contains the same cohort as Rubio-Perez et al., and showed a significantly higher potential for finding druggable targets within the spectrum of mutations harbored in these 6748 cancer patients for all three tiers (*P* < 0.0001 Z-test for all comparisons with Bonferroni correction). The improved performance is present whether we include pathway information from BioSystems in the drug recommendation or not. Therefore, the iCAGES drug score demonstrated itself to be an effective tool for predicting effective cancer therapies in multiple real-world scenarios.

## Discussion

In the current study, we established a statistical framework, iCAGES, to rapidly analyze patient-specific cancer genomic data, prioritize cancer driver events, and predict personalized therapies. Compared to currently available tools, iCAGES achieves better performance by correctly predicting cancer driver mutations, genes, and targeted drugs in analyses on both population-based cohorts and personal cancer genomes. Below we specifically discuss several unique aspects that iCAGES possesses.

iCAGES fills a practical role that other tools hardly address. While similar tools, such as MutSigCV [10] and MuSiC [11], only require genomic mutations as input, they do not allow single patient analysis due to the nature of their algorithms. Indeed, they define cancer drivers as genes whose mutation rate is significantly higher in tumors than in normal tissues among a group of patients. Since it is not feasible to calculate mutation rate in only one patient, we cannot apply these tools for personalized cancer driver prioritization, which makes iCAGES a complementary option for studying driver events for individual patients.

To facilitate researchers who only have genomic mutation data, we designed iCAGES to be less demanding on the data type. While other personalized cancer driver prioritization tools, such as DawnRank [21], often require patient’s cancer signature data, including genomic mutation, tumor gene expression data, and normal gene expression data, iCAGES only requires the patient’s genomic mutation data (in VCF format or in ANNOVAR input format) and handles all data preprocessing steps for users. Nevertheless, iCAGES users can optionally supply structural variant information (deletion/duplication), gene expression information (over/under-expressed), or cancer subtype information (for more accurate Phenolyzer analysis) to potentially improve predictions.

Another unique feature of iCAGES is that it is the only computational tool that considers point coding mutations, point non-coding mutations, and structural variants together in prioritization of driver genes. While Phen-Gen [17] also allows for point coding mutation prioritization, it ignores non-coding mutations and structural variants, which are known to contribute to cancer progression and may have a significantly different mutation pattern compared to coding mutations. Similarly, as Rubio-Perez et al. [47] pointed out, not including non-coding mutations is one of the major limitations in their work. Yet, non-coding mutations play such an important role in altering regulatory changes in the genome, as shown in recent large-scale studies such as ENCODE [77], that they are too important to neglect when prioritizing genes that drive cancer. To quantify such significance, we showed the statistical contribution of including non-coding mutations through the analysis of multivariate logistic regression of the iCAGES gene score (Additional file 9). The results show that each unit increase of the FunSeq2 score in a gene is statistically significantly associated with a 37% increase of the odds of this gene being a driver in a patient (*P* < 0.00033, two-sided Wald test).

Moreover, we would like to point out that low frequency drivers are not underrepresented in our work, in contrast to Rubio-Perez et al. We do not classify drivers based solely on their recurrences, but also consider their downstream functional impacts as well as prior knowledge on each gene’s cancer relevance distilled from decades of accumulated knowledge of gene–gene and gene–phenotype interactions. In fact, one major drawback of selecting cancer driver genes based on high mutational frequency is that all variants are weighted the same way, regardless of their downstream functional impact. We established radial SVM machine learning models that learned the patterns of cancer drivers from a set of confident and experimentally confirmed driver and passenger missense mutations to broadly prioritize cancer drivers for both recurrent and rare mutations. Instead of solely relying on mutation frequencies, our method exploits the functional impact of missense mutations and is more suitable not only for personalized cancer driver analysis but also for analysis of rare variants arising from large-scale cancer genome sequencing projects.

To better serve clinical researchers interested in personalized cancer therapy, we designed iCAGES to be one of the first tools for prioritizing personal cancer drugs. DGIdb [75] is similar in terms of this functionality because it also generates a list of drugs interacting with a list of genes. However, iCAGES’ drug prioritization layer has several advantages over using DGIdb alone. First, to enhance the specificity of drug queries, iCAGES first classifies genes into cancer suppressor genes, oncogenes, and other genes and then queries DGIdb for drugs interacting with these genes based on their role in cancer; therefore, compared to the original DGIdb drug list, the iCAGES drug list is much smaller in size and may contain fewer false positives. Second, unlike DGIdb, iCAGES prioritizes drugs using the driver potential information of a given target gene (iCAGES gene score), relatedness probability from BioSystems, and drug activity score from PubChem. Such a personalized prioritization process demonstrated itself to be not only effective in several real-world scenarios but also useful for researchers and clinicians who want to make the best use of their time and resources. Third, in some cases, DGIdb is not able to predict well-established FDA-approved targeted cancer drugs since its source databases are not updated enough to include them. To address this issue, we added an additional iCAGES-specific drug database that contains all FDA-approved targeted therapy drugs to complement the list of drugs predicted by DGIdb. Fourth, to mimic real-life scenarios where clinicians and researchers are interested in the optimal targeted drugs that are FDA approved and/or undergoing clinical trials, we also downloaded and parsed all current ongoing clinical trial information and annotated each drug in different tiers to indicate whether they are FDA-approved drugs or are undergoing clinical trials.

Another method that seems to share conceptual similarities with our iCAGES drug prioritization module is described in Rubio-Perez et al.; however, the fundamental methodologies of iCAGES personalized drug prioritization are largely different. In fact, the only overlapping method in both studies is the data source: we both refer to FDA guidelines and cancer clinical trials to search for targeted drugs. Indeed, our work attempts to cast a broader net, as we include not only drugs that have been well studied in trials and approved for market use, we also include drugs that have not been studied well but may be of interest to computational biologists and for future cancer drug screening research. Besides, a major component of our last layer is the scoring scheme, which is distinct from Rubio-Perez et al. It is true that they also included a scheme to separate predicted drugs into several tiers; however, within each tier there is no prioritization method, so that a patient may harbor multiple “driver” genes with therapies from the same tier, which is common for patients with late stage cancer and many somatic mutations. Therefore, compared to DGIdb and Rubio-Perez et al., iCAGES may be a more practical tool for personalized drug prioritization in cancer.

Furthermore, iCAGES contains a first drug recommendation pipeline that has demonstrated a high survival advantage in real-world cancer patients compared to current methods. Developing such a framework is challenging, as shown in the pioneering work by Liang and colleagues [78]. Using 953 samples from four cancer types, Liang and colleagues found that adding molecular features provides limited predictive power for predicting patient survival, which indicates the complexity of cancer molecular data and motivated us to develop a more complex architecture that can impact patient survival, from mutations, to genes, to drugs. As shown in ~25,000 cancer patients from multiple studies, iCAGES was demonstrated to be an accurate tool in each individual layer and finally a practical framework for predicting clinically relevant drug prescription. We believe that our study complements Liang and colleagues [78] in incorporating molecular, pathway, and clinical information to establish reliable therapeutic strategies.

To utilize valuable prior biological knowledge generated from numerous research studies on cancer, we integrated one of the largest biological knowledge databases on gene–gene and gene–phenotype interaction networks in the iCAGES pipeline. In its second layer, to score genes based on their prior association with cancer, we applied Phenolyzer, a database-mining tool, which integrates 15 different biological knowledge databases. Such large-scale integration of biological knowledge enhances the accuracy of iCAGES, as the prioritization process is not only based on personal genomic context but also guided by expert knowledge from decades of research.

To facilitate cancer genetics and genomics research for average biologists and clinicians, the iCAGES web server is equipped with a more user-friendly interface compared to other cancer driver prioritization tools. For example, it includes a well-documented introduction and examples so that a general user can easily learn to employ this package in his/her daily research. Moreover, to enhance the user experience, modern web technologies are employed. For example, we use the D3.js JavaScript library and render an interactive bubble plot, summarizing the outputs all in one plot (Fig. 6). For practical reasons, we also considered the time consumption of iCAGES. Using the index function embedded in ANNOVAR [25] optimized by our TDS algorithm, which outperforms Tabix [63] by ~30% for a query, the command line iCAGES achieves an average runtime of 47 s (47 ± 3.2) on a Linux cluster node with 12 CPU cores, each 2.76 GHz, for analyzing mutation data from an average patient (Additional file 1: Figure S3). We anticipate that these features of iCAGES can help average researchers without bioinformatics and machine-learning expertise to analyze genomic data, prioritize candidate genes, and search for suitable personalized therapies for a given patient.

Despite these unique advantages, as one of the first tools for comprehensive personal cancer driver and drug prioritization, iCAGES has its limitations. First, it is a challenge to obtain large-scale high quality data for training cancer driver classifiers. For example, our training dataset for the radial SVM score consists of only 939 manually curated and functionally validated missense mutations. Moreover, Cancer Gene Census may not be the ideal gold standard for classifying driver genes, as drivers in individual tumors may differ greatly due to the heterogeneity of cancer and hence may not have been included by the Cancer Gene Census database. Besides, a bias is potentially introduced by using breast cancer mutations as training data. The rationale for why we used breast cancer patients for training is because breast cancer is the most common cancer type in females and is one of the most studied cancer types with the largest amount of data from TCGA project and our model demonstrated generality in nominating cancer driver genes from 14,169 cancer patients from TCGA. Finally, to make use of information from non-coding variants from patients, we associated each non-coding variant with its genomically closest gene. This strategy is only valid for promoters, as promoters functionally explain most of the variations in RNA expression and hence downstream phenotypic changes [79]. As for other types of regulatory elements, this strategy may not be optimal. Those limitations can be overcome in the future development of iCAGES, with better training data and deeper understanding of cancer biology and regulatory elements in non-coding regions.

Last but not least, we wish to point out a caveat when using different versions of COSMIC as a database for benchmarking datasets: more stringent filtering criteria should be used in more recent versions of COSMIC data to generate more reliable testing datasets. To see whether our findings can be reproduced in other versions of COSMIC data, we compiled an additional benchmarking dataset using COSMIC version 57, with the same filtering criteria as used by the FATHMM team for training their hidden Markov model [30]. From our results, we observed similar findings as with the COSMIC version 68 dataset and excellent performance of FATHMM, as seen in their original publication. Note that the filtering criteria in version 57 is much looser than what we applied in our study; for example, to compile TP observations in the COSMIC 57 dataset, it was only required for mutations to be found in the whole gene screen and for the occurrence in the database to be greater than 5. To test whether this looser filtering strategy could be applied for compiling a testing dataset from the COSMIC version 68, we applied the same filtering criteria. We found that in this new dataset, all prioritization tools have much deteriorated performance, with an AUC around 0.5, which is almost close to random (Additional file 1: Figure S8). This result indicates that potential contamination and random noise exist in the new dataset (for example, some common germline mutations may be incorrectly classified as somatic mutations in more recent releases of COSMIC). Indeed, the major difference between these two versions of data lies in the inclusion of 1,385,270 mutations, mostly collected from large-scale sequencing projects, such as TCGA. These data made version 68 ~ 5.7 times larger than version 57. We caution that together with the amount of information from large-scale data comes extra noise, which demands a strict filtering process to ensure high quality data.

## Conclusions

We demonstrate the superior performance and effectiveness of iCAGES and we hope that this tool can complement current cancer driver detection tools, pave the way for development of such comprehensive statistical frameworks, and shed light on cancer driver gene discovery and new avenues for personalized cancer therapy.

## Additional files

Additional file 1: Supplementary information **Tables S1**, **S12**, **S14** and **Figures S1**–**S8**. (DOCX 6370 kb)
Additional file 2: Table of targeted therapy TCGA samples with complete or partial response. (XLSX 23 kb)
Additional file 3: Table of targeted therapy TCGA samples with progressive disease. (XLSX 9 kb)
Additional file 4: Table of TCGA samples with unknown response. (XLSX 27 kb)
Additional file 5: Summary of 11 point-coding variant deleteriousness prediction component scores used in the first layer of iCAGES. (XLSX 35 kb)
Additional file 6: Pair-wise Pearson correlation between all predictors and outcome variables in the training dataset. (XLSX 49 kb)
Additional file 7: Summary of backward feature selection for selecting the best set of predictors for the radial SVM model. (XLSX 54 kb)
Additional file 8: Parameter tuning for radial SVM with fivefold cross-validation on the training dataset. (XLSX 64 kb)
Additional file 9: Summary of LR model for iCAGES gene score. (XLSX 38 kb)
Additional file 10: Output of iCAGES gene score on the case study from Imielinski et al. (XLSX 46 kb)
Additional file 11: Output of iCAGES gene score on the case study from Wagle et al. (XLSX 54 kb)
Additional file 12: Output of iCAGES drug score on the case study from Imielinski et al. (XLSX 60 kb)
Additional file 13: Output of iCAGES drug score on the case study from Wagle et al. (XLSX 77 kb)

## Abbreviations

AUC: Area under curve
CI: Confidence interval
CNV: Copy number variation
CSV: Comma-separated value
iCAGES: Integrated CAncer Genome Score
LR: Logistic regression
MMAF: Maximum minor allele frequency
ROC: Receiver operating characteristic
SVM: Support vector machine
TCGA: The Cancer Genome Atlas
TDS: Two-step dictionary searching
TN: True negative
TP: True positive
