# Supplementary material for: iCAGES: integrated CAncer GEnome Score for comprehensively prioritizing driver genes in personal cancer genomes
Source: Genome Med. 2016 Dec 22;8:135. doi: 10.1186/s13073-016-0390-0 (PMC5180414; doi:10.1186/s13073-016-0390-0)
Supplement: Additional file 1: — Supplementary information Tables S1, S12, S14 and Figures S1–S8. (DOCX 6370 kb) [file 13073_2016_390_MOESM1_ESM.docx]

**iCAGES: integrated CAncer GEnome Score for comprehensively prioritizing driver genes in personal cancer genomes**

Chengliang Dong^1,2^, Yunfei Guo^1,2^, Hui Yang^1,3^, Zeyu He^4^, Xiaoming Liu^5,6^, Kai Wang^1,7*^

^1^Zilkha Neurogenetic Institute, University of Southern California, Los Angeles, CA 90089, USA

^2^Biostatistics Graduate Program, Department of Preventive Medicine, University of Southern California, Los Angeles, CA 90089, USA

^3^Neuroscience Graduate Program, University of Southern California, Los Angeles, CA 90089, USA

^4^Department of Computer Science, New York University, New York, NY 10012, USA

^5^Human Genetics Center, The University of Texas Health Science Center at Houston, Houston, TX, 77030, USA

^6^Division of Epidemiology, Human Genetics and Environmental Sciences, The University of Texas Health Science Center at Houston, Houston, TX, 77030, USA

^7^Institute for Genomic Medicine, Columbia University, New York, NY 10032, USA

***Correspondence should be addressed to:** Kai Wang, 630 W 168th St, Room 11-451, New York, NY 10032; E-mail: kw2701@cumc.columbia.edu

Table S1. iCAGES gene models for all cancer subtypes

| Subtype | Number of patients | Intercept | β for radial SVM score | β for FunSeq2 | β for CNV score | β for Phenolyzer |
| --- | --- | --- | --- | --- | --- | --- |
| ACC | 92 | 1.368 | -0.424 | -0.154 | -0.936 | -2.437 |
| BLCA | 411 | 1.059 | -0.039 | -0.004 | -0.398 | -0.909 |
| BRCA | 1094 | 1.093 | -0.076 | -0.011 | -0.480 | -1.023 |
| CESC | 298 | 1.162 | -0.149 | -0.056 | -1.327 | -1.320 |
| CHOL | 36 | -0.146 | 0.188 | 0.132 | 0.503 | 0.610 |
| COAD | 456 | -0.092 | 0.070 | 0.031 | 1.101 | 1.045 |
| COADREAD | 623 | -0.063 | 0.041 | 0.008 | 1.315 | 0.990 |
| DLBC | 48 | -0.120 | 0.139 | 0.015 | 0.538 | 0.851 |
| ESCA | 185 | -0.130 | 0.118 | 0.013 | 0.821 | 1.078 |
| GBM | 587 | -0.128 | 0.128 | 0.028 | 0.460 | 0.928 |
| GBMLGG | 1103 | -0.075 | 0.053 | -0.013 | 1.188 | 1.088 |
| HNSC | 528 | 1.058 | -0.038 | -0.005 | -0.582 | -0.946 |
| KICH | 66 | -0.123 | 0.165 | 0.103 | 0.292 | 0.465 |
| KIPAN | 887 | 1.093 | -0.074 | -0.029 | -0.345 | -1.147 |
| KIRC | 531 | 1.138 | -0.129 | -0.017 | -0.544 | -1.441 |
| KIRP | 290 | -0.075 | 0.069 | 0.017 | 1.234 | 0.968 |
| LAML | 200 | -0.060 | 0.159 | 0.027 | 0.121 | 0.024 |
| LGG | 516 | -0.116 | 0.102 | 0.045 | 0.952 | 1.111 |
| LIHC | 377 | 1.114 | -0.100 | -0.048 | -0.774 | -1.076 |
| LUAD | 571 | -0.096 | 0.074 | 0.033 | 1.462 | 1.035 |
| LUSC | 501 | 1.132 | -0.115 | -0.039 | -0.538 | -1.159 |
| OV | 590 | 1.183 | -0.175 | -0.064 | -0.726 | -1.421 |
| PAAD | 184 | 1.167 | -0.152 | -0.085 | -0.878 | -1.340 |
| PCPG | 179 | -0.126 | 0.196 | 0.063 | 0.081 | 0.290 |
| PRAD | 489 | 1.124 | -0.105 | -0.002 | -0.299 | -1.375 |
| READ | 167 | -0.130 | 0.123 | 0.030 | 1.524 | 0.990 |
| SARC | 261 | -0.077 | 0.082 | -0.007 | 0.562 | 0.772 |
| SKCM | 367 | 1.077 | -0.052 | -0.016 | -0.284 | -1.104 |
| STAD | 443 | 1.029 | -0.011 | 0.008 | -0.753 | -0.808 |
| STES | 628 | 1.029 | -0.013 | 0.0004 | -0.600 | -0.738 |
| TGCT | 150 | -0.122 | 0.140 | 0.012 | -0.014 | 0.734 |
| THCA | 507 | -0.106 | 0.126 | 0.009 | 0.754 | 0.705 |
| THYM | 123 | -0.078 | 0.155 | 0.042 | 0.257 | 0.159 |
| UCEC | 545 | -0.064 | 0.039 | 0.007 | 0.993 | 1.058 |
| UCS | 57 | -0.133 | 0.153 | -0.004 | 0.501 | 0.766 |

Table S12. Output of Phen-Gen on the case study from Imielinski *et al.*

| #GENE_ID | PROBABILITY_DAMAGING |
| --- | --- |
| OTC | 1 |
| AFF2 | 0.999112955 |
| PHKA1 | 0.990036921 |
| PAGE5 | 0.9513366 |
| UBE2A | 0.863864517 |
| **ARAF** | **0.841125607** |
| UBQLN2 | 0.666730626 |
| SSX3 | 0.514708521 |
| DRP2 | 0.424900353 |
| MAGEE2 | 0.299691661 |
| IGSF1 | 0.146688732 |
| ZC4H2 | 0.060041304 |

Table S14. Output of Phen-Gen on the case study from Wagle *et al.*

| #GENE_ID | PROBABILITY_DAMAGING |
| --- | --- |
| MAGEC1 | 0.758534246 |
| ATG4A | 0.558563519 |
| TCEAL1 | 0.307550809 |
| KLHL13 | 0.064588372 |
| RGAG1 | 0.060145623 |
| DOCK11 | 6.64E-39 |
| RPL10 | 5.67E-39 |


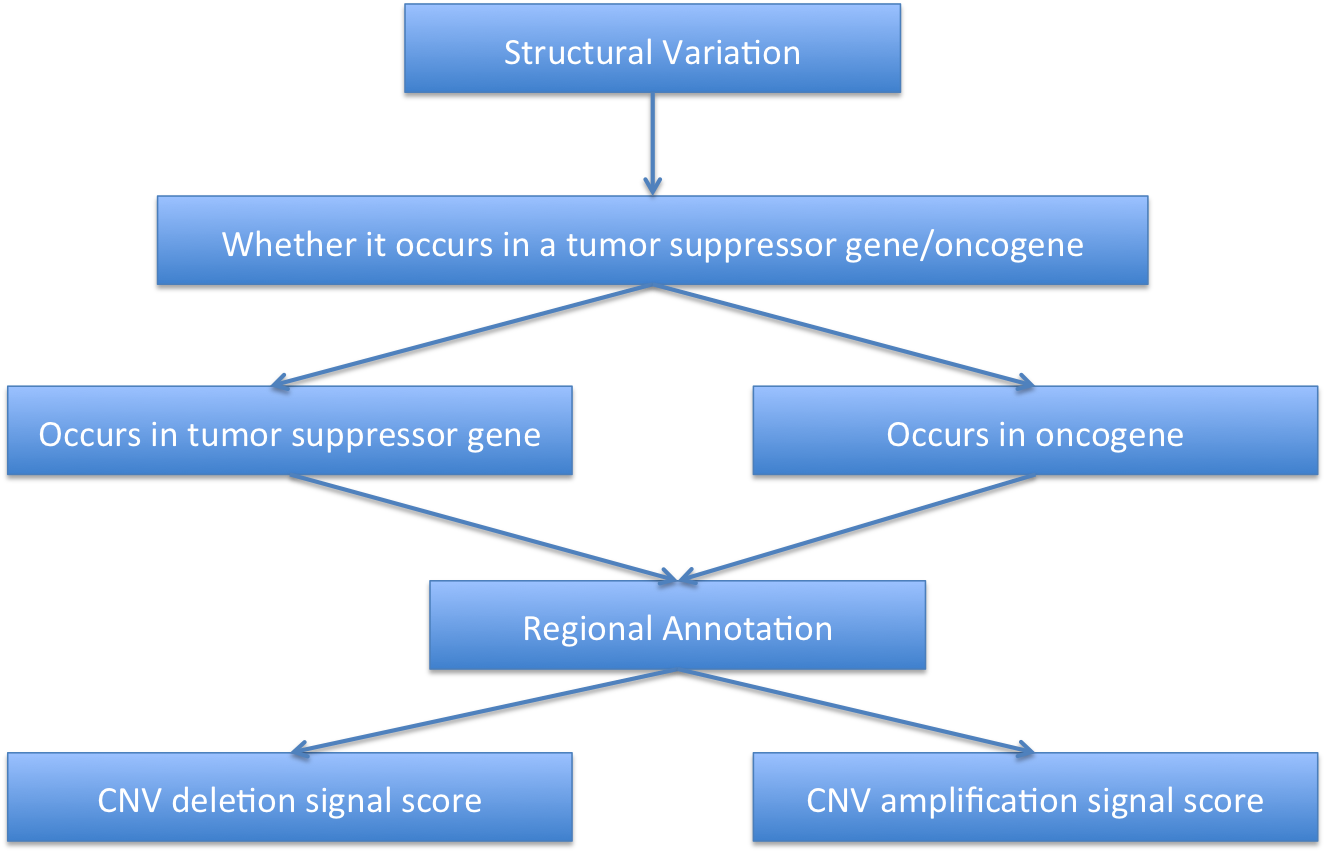


Figure S1. Flowchart of structural variation annotation module in the first layer of iCAGES. To annotate a structural variation, iCAGES first checks whether or not this variation occurs in a tumor suppressor gene or in an oncogene. If it occurs in a tumor suppressor gene or in an oncogene, then iCAGES proceed to regional annotation, using the CNV normalized deletion/amplification signal score based on Kim *et al.* and uses this as the final annotation score for such structural variation.

Figure S2. Boxplot summarizing backward model selection results for radial SVM models. The color bars indicate the AUC value of the ROC curve of the best cocktail of predictors at given number of predictors. Error bar indicates 95% CI calculated with 2,000 bootstrap replicates.


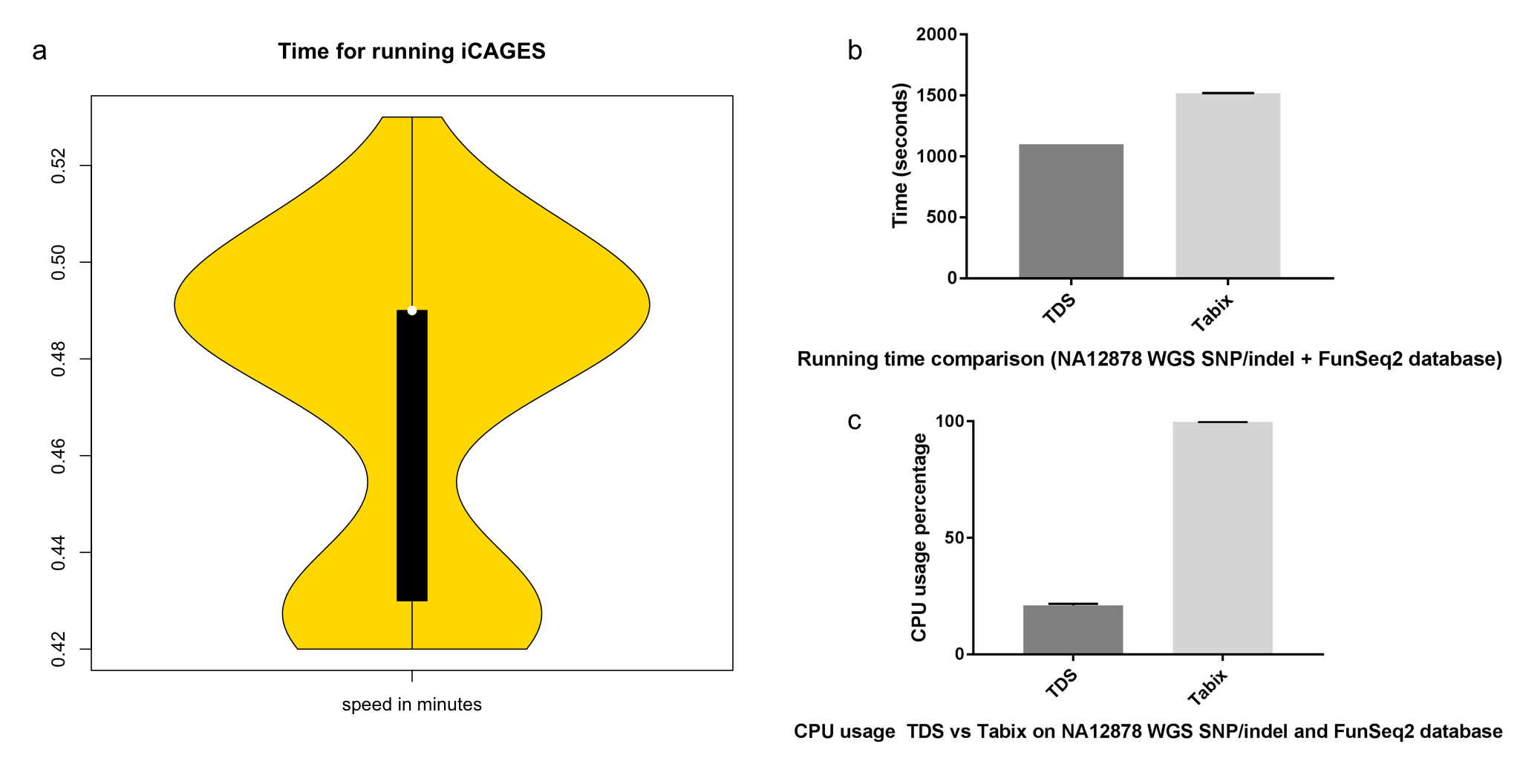


Figure S3. Speed of iCAGES pipeline. a. Violin plot of time consumption of iCAGES for running 100 patients’ data. This “violin” shows the median (indicated by the small white dot), the first through the third interquartile range (the thick, solid vertical band), and the density (shades with different colors) of the running time (in minutes) for analyzing somatic point mutations for one patient. b. Running time comparison between TDS and Tabix using NA12878. c. CPU usage comparison between TDS and Tabix using NA12878

 Figure S4. Analysis of performance of radial SVM score in the first layer of iCAGES evaluated on testing dataset with each individual score deleted for modeling. This plot illustrates performance radial SVM model implemented in iCAGES, evaluated by ROC curve and AUC score for the ROC curve. Red curve in each plot indicates a radial SVM model trained with a feature score deleted and the curve with the other color indicates the feature score being deleted and compared. Higher AUC score indicates better performance.

Figure S5. Cutoff determination using sensitivity and specificity analysis with training data set for iCAGES gene score suggests 0.11 to be ideal cutoff.

Figure S6. Analysis of performance of iCAGES gene score in the second layer of iCAGES evaluated on testing dataset with 35 cancer subtypes from TCGA cancer patients. This plot illustrates performance iCAGES gene score, evaluated by ROC curve and AUC score for the ROC curve. Red curve in each plot indicates the performance of iCAGES gene score and yellow curve indicates that of MutSigCV on the same subtype. Higher AUC score indicates better performance.

Figure S7. Analysis of performance of iCAGES gene score evaluated on the TCGA cancer data from Kandoth *et al.,* on discriminating cancer driver genes versus non-drivers. Higher AUC score indicates better performance. 95% CI was computed with 2000 stratiﬁed bootstrap replicates.

 Figure S8. Performance of mutation prioritization tools on different versions of COSMIC data. This plot illustrates performance of quantitative prediction outcomes for each predictor and two machine-learning models trained on COSMIC version 68 and version 57, both evaluated by ROC curve and AUC score for the ROC curve. Higher AUC score indicates better performance.
